# The Glucuronyltransferase *GlcAT-P* Is Required for Stretch Growth of Peripheral Nerves in *Drosophila*

**DOI:** 10.1371/journal.pone.0028106

**Published:** 2011-11-23

**Authors:** Rahul Pandey, Jorge Blanco, Gerald Udolph

**Affiliations:** Neural Development and Repair, Institute of Medical Biology, Singapore, Singapore; Virginia Commonwealth University Medical Center, United States of America

## Abstract

During development, the growth of the animal body is accompanied by a concomitant elongation of the peripheral nerves, which requires the elongation of integrated nerve fibers and the axons projecting therein. Although this process is of fundamental importance to almost all organisms of the animal kingdom, very little is known about the mechanisms regulating this process. Here, we describe the identification and characterization of novel mutant alleles of *GlcAT-P,* the *Drosophila* ortholog of the mammalian glucuronyltransferase *b3gat1*. *GlcAT-P* mutants reveal shorter larval peripheral nerves and an elongated ventral nerve cord (VNC). We show that *GlcAT-P* is expressed in a subset of neurons in the central brain hemispheres, in some motoneurons of the ventral nerve cord as well as in central and peripheral nerve glia. We demonstrate that in *GlcAT-P* mutants the VNC is under tension of shorter peripheral nerves suggesting that the VNC elongates as a consequence of tension imparted by retarded peripheral nerve growth during larval development. We also provide evidence that for growth of peripheral nerve fibers *GlcAT-P* is critically required in hemocytes; however, glial cells are also important in this process. The glial specific *repo* gene acts as a modifier of *GlcAT-P* and loss or reduction of *repo* function in a *GlcAT-P* mutant background enhances VNC elongation. We propose a model in which hemocytes are required for aspects of glial cell biology which in turn affects the elongation of peripheral nerves during larval development. Our data also identifies *GlcAT-P* as a first candidate gene involved in growth of integrated peripheral nerves and therefore establishes *Drosophila* as an amenable in-vivo model system to study this process at the cellular and molecular level in more detail.

## Introduction

During animal development and growth, the nervous system needs to expand in conjunction with the general expansion of the body. Crucial to this process is the extension of integrated nerve fibers, which contain axons connecting motoneurons in the central nervous system (CNS) to their peripheral targets, muscles and sensory neurons in the peripheral nervous system (PNS) connecting to their synaptic partners in the CNS. Neurite outgrowth, via growth-cone mediated mechanisms and growth-cone guidance, towards a target has been extensively studied [1], [2]. Current models suggest that during development axonal elongation occurs by extension of the growth cone [3]. However, once the growth cone reaches its final target and is fully connected and thus tightly integrated, growth-cone related axonal elongation mechanisms are unlikely functional any longer. Mechanical forces have been postulated to stimulate elongation of integrated axons as the animal grows [4]. This distinct process has been recently referred to as “stretch growth of integrated axon tracts” [5]. Examples of such extreme axonal stretch growth, where animal growth supplies constant mechanical tension on nerves and white matter tracts, are observed throughout the animal kingdom [6], [7]. As the anterior-posterior body axis of an animal extends during growth, the distance between most neuronal somata in the CNS and PNS and their respective target cells increases dramatically, exerting tensile forces on the axons within the nerves. These forces would normally stimulate the addition of cytoskeletal elements, axolemma and other building materials along the axon to compensate for the mechanical strain. In vitro studies have demonstrated that integrated axon tracts can undergo stretch growth [5], [8], [9]. Several studies have been reported using explanted neurons from various organisms to understand stretch induced axonal elongation in cell culture in vitro [10], [11], [12], [13], [14], [15] (reviewed in [16]). However, elongation of integrated axons is not understood at the molecular level particularly at the stage of a developing organism in vivo, partly due to the lack of mutants of genes involved in this process.

*Drosophila* offers an amenable model system to study the process of stretch growth of integrated axons, due to the high growth rate of its larval body during development as well as its compliance to genetic analyses [17]. Importantly, extensive growth is observed during the four days of larval development. During this phase, motoneuron axons that established connectivity to their muscle targets during embryogenesis have to elongate in parallel to the expansion of the larval body. However, very little is known about the cellular and molecular mechanisms controlling the extension of integrated peripheral nerves/axons during *Drosophila* larval development.

Post-translational modifications of proteins are known to be involved in nervous system development [18]. In particular, glycosylation of membrane-targeted and secreted proteins is an essential process (reviewed in [19]). Glycosyltransferases, which transfer monosaccharide units to an acceptor molecule, are responsible for the synthesis of carbohydrate moieties on proteins. The carbohydrate epitope Human Natural Killer 1 (HNK-1), found on several cell adhesion molecules such as the neural cell adhesion molecule (NCAM; [20]), is spatially and temporally regulated during development of the central and peripheral nervous systems. HNK-1 is assumed to be involved in cell-cell interactions such as cell adhesion [21], migration [22] and neurite extension [23]. The HNK-1 epitope, comprising of a unique trisaccharide structure, HSO_3_-3GlcAβ1-3Galβ1-4GlcNAc-, is sequentially synthesized by the glucuronosyltransferase P (GlcAT-P) or the glucuronosyltransferase S (GlcAT-S) and by a specific sulphotransferase (HNK-1ST) [24], [25], [26], [27], [28]. The HNK-1 carbohydrate is also crucial for maintaining proper neural function [29]. GlcAT-P glucuronyltransferase activity is also required for proteoglycan and glycoprotein biosynthesis [30], [31]. Another class of proteins modified by GlcAT-P is extracellular matrix (ECM) proteins and some ECM proteins are known to bear the HNK-1 epitope [20]. In *Drosophila*, production and secretion of several ECM molecules is an essential function of hemocytes [32]. Besides ECM deposition, hemocytes are also involved in the clearance of apoptotic cells. Possibly due to the above mentioned functions, hemocyte migration along their normal routes and arrival at their correct target tissues is vital for development and survival [32]. A single *GlcAT-P* ortholog is present in metazoan genomes, including humans, although, three paralogs have been characterized in almost all metazoans: *GlcAT-P* (also termed β-1, 3-glucuronyltransferase 1 (*B3GAT1)*), *GlcAT-S* and *GlcAT-I* [33]. GlcAT-P has been described as the predominant glycosyltransferase for HNK-1 epitope biosynthesis in the brain. Human *GlcAT-P* (*B3GAT1*) has been implicated as a candidate gene for schizophrenia-like psychosis [34] and *b3gat1* knock-out mice have been reported to have synaptic plasticity defects [35], [36]. Furthermore, *GlcAT-P* loss of function resulted in head morphogenesis defects in *Medaka* [37]. These studies together demonstrate a crucial role for *GlcAT-P* in nervous system development and/or function. However, despite the existence of a fly ortholog, very little is known about the role of *GlcAT-P* in the development and/or function of the *Drosophila* nervous system.

Here, we describe the isolation and characterization of novel alleles of the *GlcAT-P* gene which we termed *brave* (*brv*). *brv* mutants are characterized by an elongated larval ventral nerve cord (VNC). We demonstrate that VNC elongation in *brv* mutants occurs during larval development and not during embryogenesis. We show that *GlcAT-P* is expressed in a subset of central and peripheral glia, and neurons in the larval CNS. We provide evidence that in *brv* mutant larvae the peripheral nerves are shorter as compared to wild-type and as a consequence, in *GlcAT-P* mutants, the VNC is possibly under mechanical tension and most likely responds by elongation along the anterior-posterior axis. This suggests a function of *GlcAT-P* in the growth of integrated peripheral nerves during larval development. We further demonstrate that for growth of peripheral nerves GlcAT-P is critically required in hemocytes. Finally, we identify the glial specific gene *repo* as a genetic modifier of *GlcAT-P* as the VNC elongation phenotype is dramatically enhanced if the *repo* gene dose is reduced in a *brv* mutant background. Thus, we suggest that *GlcAT-P* is a major component of the genetic network required in hemocytes and glia for stretch growth of integrated peripheral nerve fibers during *Drosophila* larval development. Our work is the first to identify a gene linked to the growth of peripheral nerves.

## Results

### Isolation and genetic characterization of four novel *GlcAT-P* alleles

**Figure 1:**
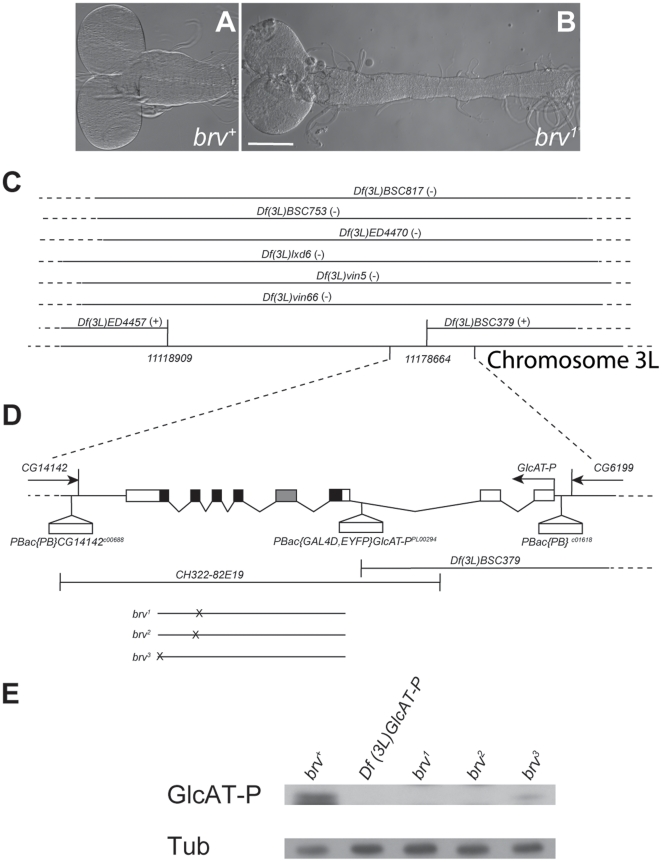
*brv* mutations are novel alleles of *Drosophila GlcAT-P.* (A, B) Phase contrast pictures of wild-type (*brv^+^*) and *brv^1^* mutant L3 larva brains showing the elongation of the larval VNC in *brv* (B). Bar: 50 µm. (C) Graphical display of the deficiencies (Df) used in the complementation test in which the chromosomal region was narrowed to 60 kb. (+), (-) indicate complementing and non-complementing Df, respectively. (D) Illustration of the structure of the *GlcAT-P* locus flanked by the predicted genes *CG14142* and *CG6199*. Arrows indicate the direction of transcription. Exons are boxed and translated regions are represented by filled boxes. The gray box indicates an exon which is spliced into one but not the other isoform. The positions in which the *brv^1^*, *brv^2^* and *brv^3^* alleles are mutated are indicated. (E) Analysis of *GlcAT-P* protein expression. Protein extracts from wild-type (*brv^+^*), *Df(3L)GlcAT-P*, *brv^1^*, *brv^2^* and *brv^3^* mutant L3 larvae were blotted with an anti-GlcAT-P antibody. Tub: α-tubulin loading control.

To identify novel genes involved in larval brain morphogenesis, we carried out an EMS screen in the genetic background of *DOPA-decarboxylase-GAL4 (DDC-GAL4); UAS-GFP* transgenic flies. In these flies, the GFP reporter protein is localized in the cytoplasm of *DDC*-expressing cells, which allows easy visualization of the first (L1), second (L2) and third instar (L3) larval CNS [38]. Three pupal lethal mutants were isolated from the screen showing a dramatically 2–3 times extended larval ventral nerve cord (VNC) as compared to the VNC of wild-type controls (Figure 1A, B). In addition to the extension in the anterior-posterior axis, the mutant VNCs also displayed a considerable reduction in circumference. Moreover, the brain lobes in *brv* mutants were oval in shape (in the anterior-posterior direction), unlike their round morphology in wild-type. Genetically, these three mutations fell into one complementation group, demonstrating that they were independent alleles of the same gene, which we termed *brave* (*brv*). Accordingly, the three mutants were termed *brv^1^, brv^2^* and *brv^3^*.

By deletion mapping, we were able to assign a region on the third chromosome spanning 10 potential candidate genes (Figure 1C) to the *brv* mutants. By sequence analysis of both the genomic DNA and the cDNAs from those 10 genes, we could demonstrate that all three *brv* alleles were single point mutations in the *GlcAT-P* gene. In *brv^1^*, a G to A transition at the splice acceptor site located immediately before the start of the 7^th^ exon resulted in a 14 bp deletion in the transcript RNA sequence due to a cryptic splicing site. As a result, a frame shift was introduced changing the amino acid sequence of the following 160 residues, before ending in a premature stop codon (see Figure 1D; Figure S1A, B). *brv^2^* was found to be a G to A transition leading to a premature stop codon at the 10^th^ amino acid of the 7^th^ exon of *GlcAT-P* (Figure S1C). Finally, *brv^3^* was characterized as a T to A transversion in the 27^th^ position of the last (8^th^) exon resulting in a non-synonymous Leu to Gln polymorphism (conserved non-polar to polar amino acid) in this position (Figure S1D, E).

To demonstrate that the observed *brv* mutant phenotype reflects a loss of *GlcAT-P* function and is not due to potential second site mutations, we followed two approaches: firstly, using the FRT containing transposons *PBac{PB}CG14142^c00688^* and *PBac{PB}^c01618^*, we generated a *GlcAT-P* null allele via flippase-induced precise deletion [39], [40]. Precise deletion of *GlcAT-P* (henceforth referred to as *Df(3L)GlcAT-P*) was verified by PCR analysis and sequencing (data not shown). In addition, either complete depletion (for *Df(3L)GlcAT-P*), *brv^1^* and *brv^2^*) or strong reduction (for *brv^3^*) of the GlcAT-P protein was demonstrated by immunoblotting using an anti-GlcAT-P antibody (Figure 1E). *Df(3L)GlcAT-P* homozygous larvae died during pupal stages and showed an extended larval VNC phenotype which was indistinguishable from the other *brv* alleles. Moreover, *Df(3L)GlcAT-P* failed to rescue the *brv^1^*, *brv^2^* and *brv^3^* mutant alleles in complementation tests. These findings suggest that *brv^1^*, *brv^2^* and *brv^3^* are functional null alleles. Secondly, a genomic construct containing exons 3 to 8 of *GlcAT-P* (P[acman] genomic construct *CH322-82E19*) rescued the larval brain defects and the pupal lethality of all three *brv* mutant alleles (Figure 1D). The dispensability of the upstream promoter region and the first two untranslated exons of *GlcAT-P* was surprising, but agreed with genetic evidence that a deficiency also lacking these elements (*Df(3L)BSC379*) genetically complemented the *brv* mutations (Figure 1D). This suggests that alternative functional promoters are present just upstream of the third exon of *GlcAT-P*. Although all 4 *brv* mutant alleles are generally pupal lethal, we found adult escapers (∼10%, n = 200) for all alleles. The escapers almost always showed developmental defects in the legs (∼98%, n = 100) and some had rudimentary wings (∼30%, n = 100).

GlcAT-P is contributed maternally (see BDGP; http://www.fruitfly.org/cgi-bin/ex/bquery.pl?qtype=report&find=CG6207&searchfield=CG). To address the function of maternally expressed GlcAT-P, we generated *brv^1^* germline clones using the FLP-DFS technique [41]. We could not detect an abnormal phenotype in the nervous system of embryos lacking both maternal and zygotic *GlcAT-P* (data not shown; see below), indicating that this gene is dispensable for embryonic nervous system development.

GlcAT-P is a key enzyme for HNK-1 epitope biosynthesis in the nervous system [42]. In mice, depletion of GlcAT-P results in an almost complete loss of HNK-1 expression in the brain [35]. On the contrary, *Drosophila Df(3L)GlcAT-P* larvae showed a similar pattern of HNK-1 modified proteins on immunoblots of brain lysates when compared to wild-type (Figure S2A). To test for possible redundancy of *GlcAT-P* and *GlcAT-S*, an anti-GlcAT-S antibody was used in immunoblots of protein extracts from *Df(3L)GlcAT-P* larval brains. GlcAT-S-specific bands were clearly detected (Figure S2A). Thus, functional redundancy of GlcAT-P and GlcAT-S might explain the presence of HNK-1 in *Df(3L)GlcAT-P* larva.

### *GlcAT-P* is expressed in a subset of glia, VUM motoneurons and mushroom body neurons

**Figure 2:**
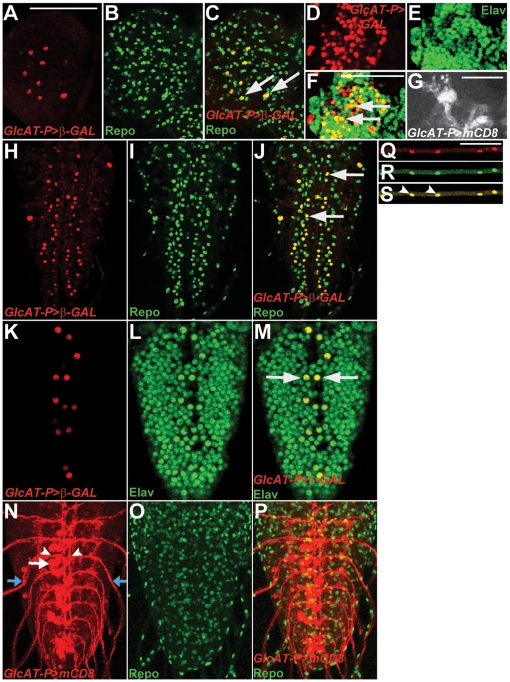
Analysis of *GlcAT-P* expression as revealed by *GlcAT-P-GAL4* driven reporter. *GlcAT-P* is expressed in the larval brain lobes (A–G), the VNC (H–P) and peripheral nerves (Q–S) in the L3 larva brain. lacZ reporter expression in the brain lobes (red, A) colabeled with α-Repo (green, B). (C) Merged frames showing that *GlcAT-P* is expressed in a subset of Repo-positive glial cells (yellow; arrows). lacZ reporter expression in the mushroom bodies (red, D) colabeled with the neuronal marker Elav (green, E). Merged frame (F) showing co-staining of GlcAT-P and Elav in Kenyon cells (yellow; arrows). (G) Membrane targeted reporter (*UAS-mCD8-mCherry*) reveals expression of *GlcAT-P* in Kenyon cell neurites. lacZ reporter expression (H, K) in the larval VNC double labeled with α-Repo (I, J) or α-Elav (L, M). *GlcAT-P* is expressed in a subset of glial cells (yellow, arrows in J). GlcAT-P expression is also found in about two Elav positive neurons in ventral midline positions (yellow, arrows in M). (N–P) *UAS-mCD8-mCherry* reporter expression reveals that two cells (white arrow in N) bifurcate (arrowheads) and extend motor axons (blue arrows) bilaterally into the periphery. (P). *GlcAT-P>lacZ* reporter expression (Q) in cells on peripheral nerves are shown. These cells are peripheral glia as they colabel with Repo (R; yellow and arrows in S). A–F, H–J and K–M show individual frames of 1 µm thickness. G and N are maximum projections of Z-stacks. Bars in A, S: 50 µm. Bars in F, G: 10 µm.

To study the expression pattern of *GlcAT-P* during larval development, we raised an anti-GlcAT-P antibody. However, we did not obtain specific signals in whole mount L3 larval brains using standard immunohistochemistry. To evaluate the functionality of the antibody, we performed immunolabeling with wing imaginal discs from transgenic larvae carrying *dpp^blink^-*GAL4 and *UAS-GlcAT-P*. A clear signal was detected along the anterior posterior border of the wing disc (Figure S2B) which was in accordance with the endogenous *dpp^blink^-*GAL4 expression pattern. Using an EYFP-tagged Golgi-specific protein (*Sqh::EYFP-Golgi*, [43]), we detected the over-expressed GlcAT-P protein in the Golgi apparatus (Figure S2C), where GlcAT-P is normally localized [44]. These findings indicate that the antibody specifically recognizes GlcAT-P protein in an over-expression paradigm in the right cellular compartment. As an alternative approach, we generated N-terminal as well as C-terminal EGFP-tagged fusion constructs of *GlcAT-P* by recombineering [45], using P[acman] genomic constructs containing the entire *GlcAT-P* locus (see methods). However, neither could we detect any specific EGFP signal in immunohistochemistry experiments, nor did the fusion constructs rescue the *brv* mutants, indicating that the EGFP tagging impairs GlcAT-P sub-cellular localization and/or function. We also attempted *in situ* hybridization experiments with GlcAT-P probes in the larval brain. However, although the probe was shown to work specifically when GlcAT-P was over-expressed in wing discs (data not shown), any *in situ* signal above background was not observed in the larval brain. Lastly, we made use of a *GAL4*-containing transposon inserted at the *GlcAT-P* locus (Figure 1D, henceforth referred to as *GlcAT-P-GAL4*). When used to drive expression of the reporter genes *UAS-nβgal* or *UAS-mCD8-mCherry*, *GlcAT-P-GAL4* showed expression in the salivary gland primordia, amnioserosa and gut during embryogenesis (Figure S3A, B; data not shown), mirroring most aspects of the endogenous in situ hybridization expression pattern (see BDGP; http://www.fruitfly.org/cgi-bin/ex/bquery.pl?qtype=report&find=CG6207&searchfield=CG). This indicates that *GlcAT-P-GAL4* recapitulates the endogenous expression pattern of *GlcAT-P*. In the larva, reporter expression was detected in the tracheal system which connects to the VNC (data not shown). *GlcAT-P-GAL4* drove reporter gene expression in the nervous system in sets of neurons in the brain lobes (Figure 2A–G), the VNC (Figure 2H–P) as well as in the peripheral nerves emanating from the VNC (Figure 2Q–S). Reporter gene expression was detected in a subset of glial cells in the brain and the VNC. In addition, GAL4 was expressed in most, but not all, of the peripheral nerve glia. Within the larval brain lobes, GAL4 was also detected in mushroom body neurons (Figure 2D–G). In the VNC *GlcAT-P-GAL4* expression was also found in two VNC neurons per neuromere (arrows in Figure 2M, N). Based on their location in ventral positions of the ventral midline, their bifurcating axons (arrowheads in Figure 2N) leaving the VNC through the peripheral nerves (red arrows in Figure 2N) and the transcriptional activity of a VUM-specific GAL4 line (*807-GAL4*, see Figure S5), these neurons were characterized as ventral-unpaired-median (VUM)-motoneurons [46], [47].

### In *brv* mutants the VNC is elongated during larval development

**Figure 3:**
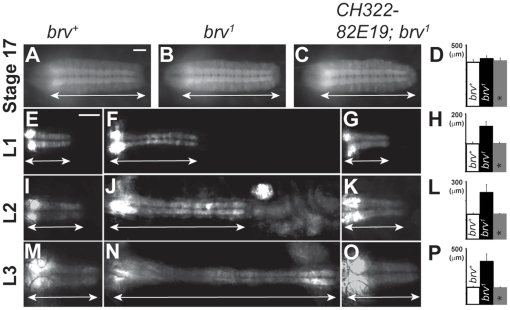
The VNC in *brv* mutants elongates during larval development and can be rescued by ectopic *GlcAT-P* expression. An *Elav-mCD8-GFP* background is used to visualize the outlines of the VNC. (A–C) Representative images of stage 17 embryos. (A) Wild-type (*brv^+^*) and (B) *brv^1^* maternal and zygotic mutant. The progressive elongation of the VNC during larval development is shown in L1 (E–G), L2 (I–K) and L3 (M–O). Larval brains from wild-type (E, I, M), *brv^1^* (F, J, N) and specimen over-expressing *GlcAT-P* (C, G, K, O) in *brv^1^* mutant background are shown. (D, H, L, P) Quantification of the length of the VNC (n = 7, for each stage) is presented (* refers to *CH322-82E19* rescued specimen. Double-sided arrows indicate the length of the VNC. L1, 2, 3 refers to larval stages 1, 2, and 3. Bars: 50 µm.

To determine when the VNC elongation occurs developmentally, we examined central nervous system development during embryogenesis and larval stages in *brv* mutants in the background of *elav-mCD8-GFP* transgenic flies [48]. During late embryogenesis (stage 17), elongation of the VNC could not be detected in maternal and zygotic *brv* mutants (Figure 3A–D). However, in *brv* mutant L1 larvae, an abnormally extended VNC was readily detectable (n = 7; Figure 3E–H). As larval development progressed to L2 (n = 7; Figure 3I–L) and L3 (n = 7; Figure 3M–P), the elongation of the VNC became more pronounced. We thus conclude that VNC elongation in *brv* mutants does not take place during embryonic development, but starts during early larval development and progresses through L2 and L3 larval stages.

**Figure 4:**
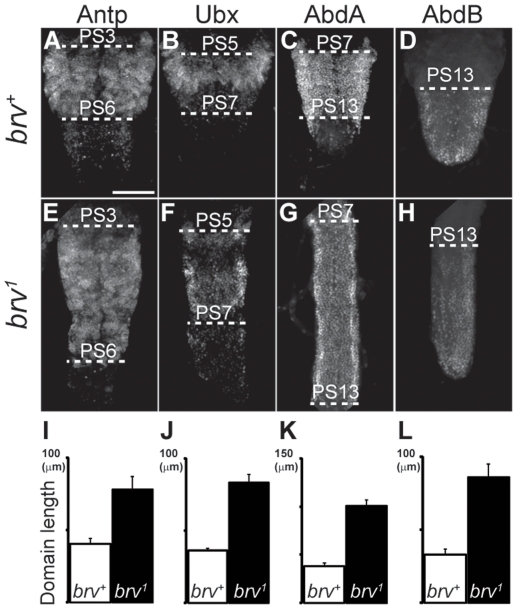
Parasegmental identity and sequence of homeotic gene expression domains are not altered in *brv* mutants. L3 larva brains from *brv^+^* (A–D) and *brv^1^* mutants (E–H) were immunostained with anti-Antp (A, E), anti-Ubx (B, F), anti-AbdA (C, G), and anti-AbdB (D, H). (I–L) Histograms comparing the average lengths of the homeotic expression domains (I: Antp; J: Ubx; K: AbdA; L: AbdB) are shown. Dashed lines indicate parasegmental (PS) boundaries. Bar: 20 µm.

To test if the sequence as well as the size of metameric units in the L3 VNC were affected in *brv* mutants, we analyzed the expression profile of the homeotic genes *Antp*, *Ubx*, *abdA* and *abdB*, which have been described to label parasegments PS3–PS5, PS5–PS6, PS7–PS12 and PS13–PS14, respectively [49]. Our results showed that all the parasegments in the *brv* mutants were present and in the correct sequence (Figure 4) suggesting that the homeotic identity of the parasegments as well as their sequence was correctly specified in the larval VNC of *brv* mutants. We checked the intactness of the VNC in *brv* mutant larvae by analyzing anti-Futsch/22C10, anti-BP102, anti-Fas2, anti-Elav and anti-Repo stainings (Figure S4). We did not find any major changes in the expression pattern of these genes and we concluded that the structural integrity of the VNC was not grossly affected in *brv* mutants.

We also checked the integrity of mushroom body neurons and VUM neurons in *brv* mutant larval brains by labeling them with *UAS-mCD8-GFP* using *GAL4-OK107* [50] and *807-GAL4* (A. Brand, unpublished), respectively. The *brv* mutation did not affect the viability of these neurons, but a displacement of their cell bodies and neurites along the longitudinal axis was observed (Figure S5) possibly as a consequence of the elongation of the nervous system along the anterior-posterior axis.

### Neuroblast proliferation is normal in *brv* mutants

**Figure 5:**
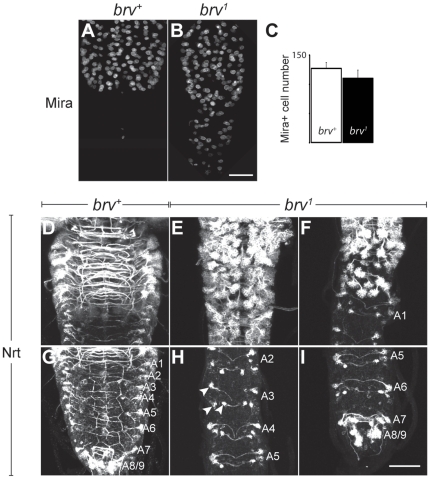
Larval VNC extension is not a consequence of extra larval NB and GMC proliferation. (A–C) Comparison of progenitor cells (neuroblasts and GMCs) in the Antp expressing domain (anti-Antp staining not shown) of (A) *brv^+^* and (B) *brv^1^* mutants using anti-Mira. (C) Quantification of Mira positive cells in *brv^+^* and *brv^1^*. (D–I) anti- Nrt staining in thoracic (D–F) and abdominal neuromeres (G–I) of L3 larva brains in *brv^+^* (D, G) and *brv^1^* mutants (E, F, H,I). A1–A9: abdominal neuromeres 1 to 9. Arrowheads in H point to the 3 Nrt positive NB lineages in A3. A–B and D–I represent maximum projections of Z-stacks. Bar: 20 µm.

To examine whether the extension of the VNC could be due to additional cells, we studied neuroblast (NB) proliferation in *brv* mutants. We firstly analyzed the number of proliferating cells in thoracic and abdominal segments of *brv* mutant VNCs during L3 larvae by anti-Miranda staining. Miranda (Mira) labels dividing NBs and ganglion mother cells (GMCs). We could not detect any significant difference in the number of proliferating Mira positive cells in *brv* mutant versus wild-type larval brains (n = 6; Figure 5A–C). We also analyzed neuroblast lineages in the larval VNC of L3 *brv* mutants using anti-Neurotactin (Nrt) immunohistochemistry. In L3 larva, Nrt labels active NBs and their larval-specific secondary neurons [51]. Extra Nrt positive cells indicative of extra proliferation could not be detected in thoracic segments (Figure 5D–F) or in abdominal segments (Figure 5G–I) of the VNC. For example, in wild-type VNCs, only 3 NBs per hemisegment are mitotically active during L3 stages producing small lineages that contain 4 to 12 Nrt positive cells [52]. Both the number of Nrt labeled NB lineages and the number of cells within these lineages was comparable between wild-type and *brv* mutant abdominal neuromeres (Figure 5G, H–I). However, as a consequence of the VNC extension, the spacing between the abdominal Nrt positive lineages was increased in *brv* mutant larvae (Figure 5H, I). In the thoracic segments, the extension resulted in a misplacement of NB lineages towards the midline of the VNC, a domain reserved to the neuropile in wild-type brains. In summary, we present evidence that the elongation of the VNC in *brv* mutant larvae is not due to extra cell proliferation of larval NBs.

### Peripheral nerves are shorter in *brv* mutant larvae

**Figure 6:**
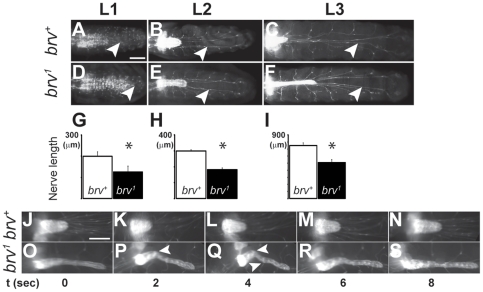
*brv* mutant larvae display shorter peripheral nerves. Representative pictures of L1, L2 and L3 larval brains from *brv^+^* (A, B, C) and *brv^1^* mutants (D, E, F) expressing *Nrv2-GAL4;UAS-GFP^(S65T)^* to visualize the peripheral nerves. White arrowheads indicate the most posterior projecting and longest peripheral nerves used for these measurements. Histograms (G, H, I) compare the average lengths of the longest peripheral nerves during L1 (G), L2 (H) and L3 (I) larva between wild-type and *brv^1^* mutants. The peripheral nerves are significantly shorter in *brv^1^* mutants (*: p<0.0001). (J–S) Representative frames, taken from time-lapse recordings of wandering larva expressing *Nrv2-GAL4; UAS-GFP^(S65T)^*, display the VNC during one full cycle of a larval contraction and expansion during locomotion. A *brv^+^* (J–N) and *brv^1^* larva (O–S) is shown. Ripples (arrowheads in P, Q), indicative for relaxation from tension, were detected only in VNC of *brv^1^* mutants but not in *brv^+^* (J–N). Bar: 100 µm. A time line (in sec) is shown below panels J–S.

Since *GlcAT-P-GAL4* reporter gene expression was found in the peripheral nerves and in the VUM motoneurons whose axons project through the peripheral nerves towards their peripheral muscle targets (Figure 2K, N, Q), we investigated whether the length of these peripheral nerve fibers was affected in *brv* mutant larvae. Using *Nervana2-GAL4 (Nrv2-GAL4)*; *UAS-GFP* transgenic flies [53], we measured the length of the longest nerve fibers emanating from the most posterior end of the VNC in *brv* mutants and compared it to wild-type. A substantial difference in the length of the peripheral nerves was already detectable during L1 stages but not at stage 17 embryos (data not shown) and the difference was more evident in L2 and L3 larva (Figure 6A–I). We found that nerve fibers in *brv* mutants were considerably shorter (wild-type: 750+/- 36 µm; *brv* mutants: 508+/- 40 µm, n = 7 for each) (Figure 6C, F, I) at L3 stage. The data demonstrated that consistently peripheral nerves were shorter by about one third in *brv* as compared to wt. As a consequence of the nerve fibers being shorter, they also contained less total peripheral glia as compared to wild-type, although the spacing and the distribution of peripheral nerve glia on a similar long stretch of the nerve fibers did not seem to be affected (Figure S6).

**Figure 7:**
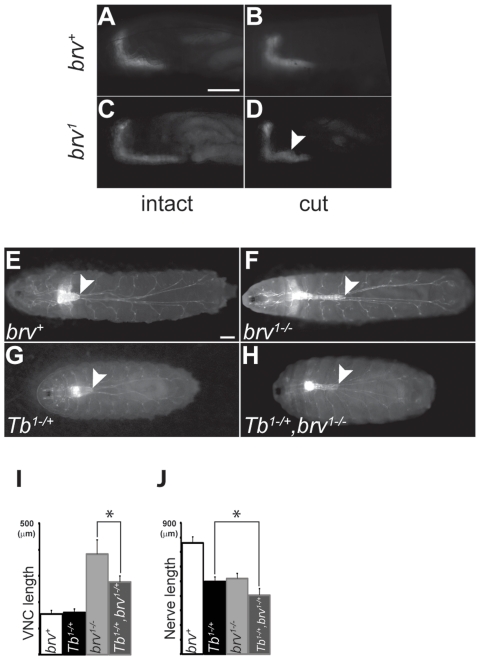
In *brv* mutant larvae the VNC appears to be under tension of peripheral nerves. Representative pictures of L2 larvae from *brv^+^* (A, B) and *brv^1^* mutants (C, D) expressing *Elav-mCD8-GFP* before (A, C) and after (B, D) dislodging the connections of the posterior most peripheral nerves. Arrowhead in (D) points towards a kink that appeared during relaxation of the VNC in *brv^1^* mutants. Representative pictures from *brv^+^* (E), *brv^1-/-^* (F), *Tb^1-/+^* (G) and *brv^1-/-^*, *Tb^1-/+^* mutant (H) L3 larvae expressing *Nrv2-GAL4;UAS-GFP^(S65T)^*. The e\tension of the VNC in *brv^1-/-^*, *Tb^1-/+^* is reduced as compared to *brv^1-/-^*. Histograms (I, J) quantify the average lengths of VNCs and posterior-most peripheral nerves. *brv^1-/-^, Tb^1-/+^* VNCs are significantly shorter as compared to *brv^1-/-^* mutants (*: p = 0.0005). White arrowheads indicate the posterior tip of the VNC. Bars: 100 µm.

Shorter peripheral nerves could either be causative for the extension of the VNC or, alternatively, a consequence of the elongated VNC. To resolve this issue, time lapse recordings of wandering L3 wild-type and *brv* mutant larvae were performed. In wild-type larvae, the brain and VNC as well as the peripheral nerves were rapidly stretched and relaxed as the larvae moved (Figure 6J–N; see also [53]). However, stretching and compression was more evident in *brv* mutant larvae, in which the VNC contracted more strongly and developed ripples as the larvae constricted (arrowheads in Figure 6O–S). This suggested that the larval VNC was under higher tension in *brv* mutants. To further investigate whether the VNC is indeed under tension of shorter peripheral nerves, we made use of *elav-mCD8-GFP* transgenic flies [48] and observed the VNC in real time in wild-type and *brv* mutant L2 larvae. Larvae were cold-immobilized and a cut was made at the posterior end. This cut severed the connections between the most posterior peripheral nerve fibers and their respective body wall contacts and as a consequence any tension of the most posterior nerves on the VNC would be released. We observed that after this cutting procedure the VNC immediately retracted in *brv* mutants (n = 10; Figure 7C, D), whereas there were no detectable changes in wild-type larvae (n = 10; Figure 7A, B). VNC retraction could not be seen in *brv* mutant L3 larvae after an identical procedure, possibly because during L3 larval stages the VNC is too deformed to return to its original length. To further resolve if VNC elongation is the cause or the consequence of shorter peripheral nerves, we made use of the *Tubby (Tb*) mutant. *Tb* larvae are considerably shorter along the anterior-posterior axis than *wild-type* larvae [13]. Although the length of the *Tb* VNC was comparable to *wild-type* (Figure 7G, I), peripheral nerves were shorter in *Tb* larvae (Figure 7G,J). However, when we compared VNC extension in *brv^-/-^* and in *brv^-/-^; Tb^-/+^* mutant L3 larvae, we found that in *brv^-/-^; Tb^+/-^* mutants the VNC was also extended but significantly lesser as compared to *brv^-/-^* mutants alone (Figure 7E–I). This experiment demonstrated that the extension of the VNC in *brv* mutants correlates with the total length of the larva. Since the peripheral nerves and the tracheal system are the only physical connections between the larval body wall and the CNS, our results suggested that in *brv* mutants the VNC expands as a consequence of increased tension from shorter peripheral nerves or trachea possibly due to some growth retardation of larval peripheral nerves or trachea.

### GlcAT-P function is required in hemocytes

To test for the cell specific requirement of GlcAT-P, rescue experiments with ubiquitous (*Act5C-GAL4)* as well as cell specific GAL4 drivers for the tracheal system (*btl-GAL4)*, neuronal (*Elav-GAL4)* and glial cells (*gcm-GAL4* and *repo-GAL4*) were performed. We were unable to rescue *brv* mutants with the *btl-GAL4* tracheal driver ruling out any involvement of the tracheal system at least in the VNC extension phenotype. We also could not rescue *brv* mutants with the pan-neuronal driver *Elav-GAL4* demonstrating that expression of *GlcAT-P* in neurons is not sufficient to rescue the VNC extension phenotype. However, ubiquitous expression by *Act5C-GAL4* completely rescued the phenotype (Figure S7; n = 50) showing that the VNC extension in *brv* mutants is a specific consequence of loss of GlcAT-P function. Furthermore, the catalytically dead version of GlcAT-P (GlcAT-P^cd^) driven by *Act5C-GAL4* failed to rescue demonstrating that impairment of GlcAT-P catalytic activity is sufficient for the *brv* mutant phenotype. GlcAT-P driven by the pan-glial and hemocyte driver *gcm-GAL4* fully rescued the extended VNC phenotype as well as the pupal lethality (Figure S7; n = 50). However, another pan-glial driver, *repo-GAL4*, was not able to rescue the *brv* mutant phenotype (Figure S7; n = 50). Most importantly, the *brv* mutant phenotype was completely rescued (Figure S7; n = 50) when GlcAT-P was driven by the hemocyte specific driver, *crq-GAL4*. The rescue experiments identified a critical role for GlcAT-P in hemocytes.

### *repo* is a genetic modifier of *GlcAT-P*

**Figure 8:**
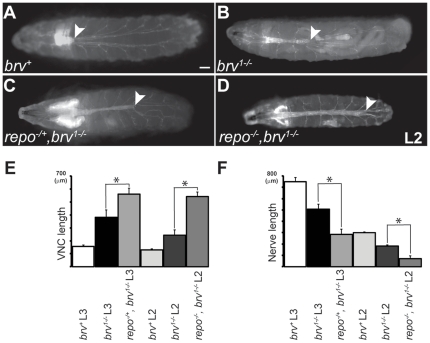
*repo* interacts genetically with *GlcAT-P.* Representative pictures from *brv^+^* (A), *brv^1^* (B), *repo^-/+^, brv^1-/-^* L3 (C) and *repo^-/-^, brv^1-/-^* L2 larvae. The CNS is visualized either by *Nrv2-GAL4;UAS-GFP^(S65T)^* (A, B) or by *repo-GAL4;UAS-GFP^(S65T)^* (C–D). White arrowheads indicate the tip of the VNC. (E, F) Histograms compare the average lengths of VNCs (E) and the most posterior peripheral nerves (F) for L3 as well as L2 larvae. *repo^-/+^, brv^1-/-^* VNCs are significantly longer as compared to *brv^1-/-^* mutants (*: p<0.0001). Bar: 100 µm.

In an attempt to identify interactors with *GlcAT-P*, we tested a possible genetic interaction with the glial specific *repo* gene. Our first observation that *repo* and *GlcAT-P* interacted came from our rescue experiments when we observed enhanced elongation of the VNC in the presence of one chromosome carrying the lethal *repo-GAL4* (henceforth referred to as *repo*) insertion in the background of *brv^1^* ^-/-^ larvae (Figure S7). The length of the extended VNC in *brv^1-/-^*, *repo^-/+^* L3 larvae was 560.8+/- 44.8 µm as compared to 382.8+/- 54.8 µm in *brv^1-/-^* mutant larvae (Figure 8A–C, E; n = 7 for each). This difference was statistically significant (p<0.0001). Correspondingly, the posterior-most peripheral nerves shortened from 508.6+/- 40.7 µm in L3 *brv^1-/-^* mutant larvae to 283.3+/- 48.8 µm in *brv^1-/-^*, *repo^-/+^* larvae (Figure 8A–C, F; n = 7 for each). The shortening of peripheral nerves is statistically significant (p<0.0001). Furthermore, rare *brv^1-/-^*, *repo^-/-^* L2 escapers showed an even more severe extension of the VNC as compared to *brv^-/-^* L2 larvae and a similar extension as *brv^-/-^*, *repo^-/+^* L3 larvae (Figure 8D, E). The increase in *brv^1-/-^*, *repo^-/-^* L2 larvae was also significant (p<0.0001) compared to L2 *brv^-/-^* larvae. It is worth noting that the VNC of *brv^1-/-^*, *repo ^-/-^* L2 larvae almost spanned the entire length of the larva (Figure 8E; see also Figure 3L), a situation similar to late embryos where the VNC extends to the most posterior end of the embryo. Interestingly, the peripheral nerves were also severely shorter in L2 larvae suggesting that growth of peripheral nerves came to an almost complete halt in *brv^1-/-^, repo^-/-^* double mutants (Figure 8F). These results strongly support the notion that hemocytes as well as glial cells are synergistically required for the growth of integrated peripheral nerves during larval development.

Taken together, our data demonstrate that VNC extension in *brv* mutants may be a result of increased tension exerted on the VNC as a consequence of impaired peripheral nerve extension/growth. In this context, *GlcAT-P* plays a crucial role in hemocytes which might control some aspect of glial cell biology implicated in the growth of integrated peripheral nerves. Further studies are required to address the exact molecular and cellular mechanisms of *GlcAT-P* function in hemocytes and glia.

## Discussion

In this study, we have identified and characterized novel alleles of the glucuronyltransferase *GlcAT-P*, the *Drosophila* homolog of human *B3GAT1*. The loss of *GlcAT-P* function causes an extension of the VNC probably due to the inability of the peripheral nerve to stretch/grow in tandem with the growth of the larval body. We also showed that hemocytes are critically required for the extension of peripheral nerves during larval development but glia also plays an important role. Our findings suggest that *GlcAT-P* is involved in growth of integrated peripheral nerves during *Drosophila* larval growth.

### *GlcAT-P* is expressed in the larval nervous system

Although we spent considerable effort to study GlcAT-P protein localization in larval brains, we were unsuccessful in detecting any relevant signal using an antibody raised against *Drosophila* GlcAT-P in tissue whole mounts although the antibodies worked on western blots and in an over/mis-expression paradigm. The fact that we could not detect *GlcAT-P* expression in specimen could be attributable to a low endogenous expression level of *GlcAT-P* or, alternatively, to steric hindrance of the antigenic epitope(s) due to the particular subcellular localization of the GlcAT-P protein or to its interaction with other proteins. However, a *GlcAT-P-GAL4* line with an insertion into the *GlcAT-P* locus allowed us to study the embryonic and larval expression pattern of *GlcAT-P*. *GlcAT-P-GAL4* driven LacZ reporter expression could not be detected in the hemocytes or embryonic CNS. But LacZ expression was clearly detected in a subset of neurons (Kenyon cells and VUM motoneurons) and glial cells in the larval CNS. The *brv* mutation neither impaired the viability of these neurons nor resulted in obvious developmental defects, apart from the longitudinal displacement of their cell bodies and neurites due to VNC elongation.

During *Drosophila* nervous system development, the VNC undergoes a process of condensation at late embryonic stages [54]. Recently, a mutation in the *C1GalTA* gene, which codes for a core 1 galactosyltransferase, has been isolated and its phenotype characterized as a defect in embryonic VNC condensation [55]. *C1GalTA* is one of the several *C1GalT*s genes involved in mucin-type *O*-glycosylation in *Drosophila* [55]. Interestingly, the *C1GalTA* mutant phenotype is almost indistinguishable from *brv* mutants with regard to the larval VNC extension. However, we have not detected embryonic VNC condensation defects in *brv* mutants. We attribute this difference to the expression of *C1GalTA*, but not *GlcAT-P*, in the embryonic CNS [55].

### *GlcAT-P* controls the length of the VNC and the peripheral nerves

We observed a reverse correlation between the length of peripheral nerves and the extension of the VNC in *brv* mutants. It has been reported that tension on nerves during growth might stimulate the elongation of axons [5]. It is conceivable that if tension on nerves generated by the growing larval body cannot be counteracted by concomitant growth of the peripheral nerves, the VNC would be forced to compensate such tension by stretching, thus resulting in the VNC extension phenotype observed in *brv* mutants. Such notion is supported by the retraction of the VNC in *brv* mutant larvae after severing the very posterior segments. This procedure liberates the attachment of the most posterior peripheral nerves to the posterior segments of the larva and at least partially releases the tension on the nerves and the VNC. Thus, our data is consistent with the hypothesis that *GlcAT-P* plays a role in peripheral nerve growth during *Drosophila* larval development. Alternatively, GlcAT-P might be required in the VNC during larval stages to keep the VNC compacted. However, our finding that the VNC extension is significantly reduced in *brv^-/-^, Tb^+/-^* mutants as compared to *brv^-/-^* mutants indicates that the VNC is not undergoing an active extension in *brv* mutants and that the elongation is clearly dependent on the total length of the larval body. This observation suggests that GlcAT-P is not required for VNC compaction since the VNC should have been elongated to a similar extent in the *Tb^+/-^* background if GlcAT-P would function in VNC compaction. Nevertheless, it cannot be completely ruled out that *brv* mutation causes enhanced VNC elasticity due to changes in the ECM deposited into the VNC by hemocytes and maybe glia. As a consequence of reduced VNC rigidity and its extension, the peripheral nerves do not bear significant strain and hence are not induced to grow. It is also feasible that both a defect in peripheral nerve growth as well as in the elasticity of the VNC contribute to the extension of the VNC. However, even in the context of the alternative explanation it is still obvious that the final consequence is that peripheral nerves in *brv* mutants do not elongate as much as they would in the *wild type* (*wt*). During larval growth the terminals of axons in the peripheral nerves progressively move away from the nervous system predominantly as a consequence of the extension of the larva along the anterior-posterior body axis. During *wt* larvae growth the length of the VNC does not elongate much whereas the peripheral nerves increase quite dramatically in length. This reveals a mechanism which promotes extension of integrated peripheral axons during larval growth. In contrast, in *brv* mutants a significant reduction of the length of peripheral nerves is observed and we suggest that due to the reduced growth of peripheral nerves the VNC compensates the resulting tension by elongating. Taken together, we conclude that *GlcAT-P* controls the elongation of peripheral nerves/axons either indirectly via regulating the compaction of the VNC or by directly regulating the growth of peripheral nerves and the axons therein.

It is worthwhile to note that most organisms with a nervous system exhibit elongation of the anterior posterior body axis during development which normally goes beyond the growth of the nervous system along the same axis. The contacts between e.g. centrally located motoneurons and their peripheral muscles are generally established before such extensive growth phases. A mechanism of “stretch growth of integrated axon tracts” [5] has been postulated to allow peripheral nerves and the integrated axons in these nerves to grow in order to compensate for possible tension arising from the growth process of the organism on the central nervous system. Although such stretch growth of integrated axon tracts seems to be fundamental to all growing organisms, almost nothing is known about the cellular and molecular mechanisms driving this process. Our data suggests that GlcAT-P is involved in this mechanism as in the absence of GlcAT-P function peripheral nerve growth is severely retarded.

### GlcAT-P may regulate growth of integrated nerves via glia and hemocytes

We also have demonstrated that GlcAT-P function is important in hemocytes since targeted expression of GlcAT-P in hemocytes fully rescued the VNC extension as well as pupal lethality whereas neither expression in neurons nor in glia could rescue. Hemocytes are known as versatile cells involved in immune response as well as in immune system unrelated developmental mechanisms [32]. Interestingly, hemocytes have been shown to be required for brain morphogenesis particularly in the process of embryonic VNC condensation [54]. The onset of VNC condensation coincides with the deposition of Collagen IV by hemocytes and a lack of hemocyte migration is associated with a severe reduction in ECM components [54] demonstrating that one major function of hemocytes is ECM deposition. This is further supported by findings that hemocytes express several ECM components such as Peroxidisan, dSPARC, and the proteoglycan MDP; also it has been shown that hemocytes produce structural components of the basement membrane such as laminin A and collagen IV [56], [57]. GlcAT-P could have a role in modifying ECM components produced in hemocytes by adding sugar residues to such proteins which in turn might influence cells directly exposed to such modified ECM.

We have also shown that partial or complete loss of *repo* function in *brv* mutant background results in further and progressive extension of the VNC depending on the *repo* gene dose. These observations clearly reveal that although GlcAT-P function is required in hemocytes, the phenotypic manifestation of VNC extension in *brv* mutants also involves functional glial cells. *repo* affects glia differentiation as well as maintenance of glial function [58], [59], [60]. Our finding indicates that if the population of glial cells is genetically weakened by reducing or removing Repo function in a *brv^-/-^* mutant background, the VNC extension phenotype is enhanced. Strikingly, in specimen double mutant for *brv* and *repo*, the extended VNC stretches along the entire body axis of the larva. This is comparable to the VNC extension in late embryos suggesting that in *brv^-/-^, repo^-/-^* double mutants, the growth of peripheral nerves is almost completely abolished thus leading to the extreme extension of the VNC during larval growth. Furthermore, the genetic interaction between *brv* and *repo* establishes an important role for glia in stretch growth of peripheral nerves and/or VNC elasticity. Interestingly, the peripheral nerves are covered by a thick layer of ECM [61]. As such, the ECM might be an important provider of contextual information for peripheral glia as well as neurites projecting through the nerves.

Some peripheral glial cells also express *GlcAT-P* which might suggest that glial cells are also involved in either generating their own matrix environment or directly control neurite extension. However, expression of GlcAT-P specifically in glial cells did not rescue the *brv* mutant phenotype and as such GlcAT-P function in glia is not critical for peripheral nerve growth. Thus, we hypothesize that GlcAT-P has a role in regulating the elongation of integrated nerves and peripheral axons during development via hemocytes and glia possibly by glycosylating and depositing ECM related proteins. GlcAT-P which is mainly expressed in the brain in vertebrates [30] has been shown to be involved in the biosynthesis of the HNK-1 epitope [26] on integral membrane glycoproteins such as the neural cell adhesion molecule [62], myelin-associated glycoprotein [63], L1 [62], transiently expressed axonal glycoprotein-1 [64], P0 [65] and Ependymin [66]. In addition, some proteoglycans and glycolipids are also known to bear the HNK-1 epitope [20], and in mice, the HNK-1 epitope is selectively found on myelinating Schwann cells associated with motor axons [19]. In *GlcAT-P*/*b3gat1* knock-out mice, an almost complete loss of the HNK-1 epitope in the brain is observed [35]. In our analysis the HNK-1 epitope was still detected in immunoblots of *brv* mutant larval CNS, suggesting that in the fly, GlcAT-P is not required or is redundant for the generation of the HNK-1 epitope. Indeed, GlcAT-S has been shown to be involved in HNK-1 epitope synthesis as well [24] and we have demonstrated that GlcAT-S protein is present in the fly larval-CNS. Therefore, in *Drosophila* at least a partial redundancy between *GlcAT-P* and *GlcAT-S* might explain the overall presence of the HNK-1 epitope. It is worth noting that despite the apparent lack of changes in global levels of the HNK-1 epitope, the VNC extension phenotype was still completely penetrant in *brv* mutant larvae. Hence, the extended VNC phenotype in *brv* mutant larvae could be independent of the HNK-1 epitope. This is also supported by the similarity to the phenotype of *C1GalTA* which might indicate that *GlcAT-P*, like *C1GalTA*, is also involved in generation of glucuronylated core 1 O-glycans [67], [68]. Alternatively, our analysis of HNK-1 expression levels might have overlooked a specific HNK-1 carrying protein responsible for the VNC extension phenotype, and as such VNC extension could still be a result of a specific, yet unknown, protein lacking the HNK-1 epitope as a consequence of the loss of GlcAT-P activity. All three *brv* alleles identified in the EMS screen affected the C-terminal catalytic domain of GlcAT-P and ectopic expression the GlcAT-P^cd^ was not able to rescue *brv* mutants. We conclude that loss of catalytic GlcAT-P activity is sufficient to result in a functional null mutation and the catalytic activity of GlcAT-P is crucial for peripheral nerve growth.

Interestingly, one of the currently identified GlcAT-P protein targets in vertebrates is the HNK-1 epitope carrying ECM glycoprotein tenascin-C [67]. Ten-C is found in glial cells and potentially promotes or inhibits neurite outgrowth in a context dependant manner [69], [70]. Glial cells and neurons/axons are also known to interact with each other [71] by glia supporting axonal functions e.g., by neurotrophic and metabolic support [72] as well as by providing the extracellular matrix context [69], [70]. Based on these reports and our own observations, we suggest a model in which GlcAT-P is critical in hemocytes possibly to deposit specific ECM on peripheral nerves. The ECM then in turn is critical to peripheral glia for stretch growth of the peripheral nerves for example by directly or indirectly being involved in the incorporation of new membrane material to growing axons thereby facilitating stretch growth of integrated axons during larval growth. It has been shown that ECM is more than a mere structural support to cells and instead is involved in signaling on the molecular level via ECM receptors such as integrins, dystroglycans, membrane associated heparan sulphate proteoglycans (HSPGs), glycipicans and syndecans [73], [74], [75]. For example, integrins are crucial components of cell adhesion and together with other ECM receptors can signal to the cytoskeleton of cells leading to cell shape changes, regulation of cell proliferation, cell migration, apoptosis and differentiation. Furthermore, a clear link between ECM and axonal growth and growth cone pathfinding has been established [73]. In this context the ECM has been shown to regulate actomyosin contractility, actin polymerization and actin stabilization and therefore might impact on the cytoskeleton of signaling receiving cells via Src, FAK, Rac1, RhoA and Rock leading to modified growth cone pathfinding [76], [77]. Therefore, it is possible that in the context of the extended ventral nerve cord and the shorter peripheral nerves in *GlcAT-P* mutant larvae altered ECM deposited by *GlcAT-P* deficient hemocytes and glia might lead to profound changes in the ECM to cell signaling resulting in the observed peripheral nerve growth phenotype. It is conceivable that ECM related mechanisms contributing to early growth cone pathfinding are also involved in growth of axons after the growth cones reached their final target sites. However, currently the precise molecular and cellular mechanisms how *GlcAT-P* affects axonal extension of integrated axons in *Drosophila* peripheral nerves are unknown and need further investigations.

Alternatively, GlcAT-P could play a role in glial proliferation or migration. This finds some support by the observation that at least the peripheral nerves had less total glia although spacing and distribution of glial cells along the nerves seemed to be unperturbed. The limited reduced number of peripheral glia could then hamper the extension of peripheral nerves and thus limit the elongation of peripheral axons. However, we did not observe a general reduction of glial cells in *brv* mutants making a general role of GlcAT-P in glial proliferation relatively unlikely.

In summary, we have isolated novel alleles of the *Drosophila* homolog of the mammalian glucuronyltransferase *b3gat1*, *GlcAT-P.* Functional analysis suggests that GlcAT-P is required in hemocytes for the growth of peripheral nerves during development in which glial cells also play a major role. The postembryonic growth of organisms requires the continuous elongation of integrated peripheral nerves and axons to accommodate for the extension of the body axes during development. To our knowledge, *GlcAT-P* is the first gene with a possible link to nerve stretch growth. We propose that the developing *Drosophila* larval nervous system and the *GlcAT-P* mutants characterized here will be helpful in deciphering the basic cellular and molecular mechanisms of nerve stretch growth in the future. Currently, the major challenge is the identification of potential *GlcAT-P* targets either via biochemical or genetic screens and the characterization of their functional involvement. Given the high degree of functional conservation of molecular and biochemical pathways between insects and vertebrates, performing further mechanistic studies on GlcAT-P in *Drosophila* most likely will deliver new insights into the roles of glucuronyltransferases in brain development and function.

## Materials and Methods

### Fly stocks

w*; DDC-GAL4 [38] and w*; UAS-GFPS65T were meiotically recombined on the third chromosome. For the EMS mutagenesis screen w*; DDC-GAL4, UAS-GFPS65T males were fed with 25 mM EMS according to standard procedures. Following this, they were mated with w*; Dr^1^/TM6b, Dfd-GMR-nv-YFP, Sb^1^, Tb^1^ virgin females. Single males from the progeny were then mated with w*; Dr^1^/TM6b, Dfd-GMR-nv-YFP, Sb^1^, Tb^1^ virgin females to propagate the mutagenized third chromosome. Finally, w*; DDC-GAL4, UAS-GFPS65T ^#^/*Dr^1^/TM6b, Dfd-GMR-nv-YFP, Sb^1^, Tb^1^* males and female virgins were mated together to establish the stocks (^#^ denotes mutagenized chromosome). *DDC-GAL4, UAS-GFPS65T* was meiotically recombined out of *brv^1^*, *brv^2^* and *brv^3^* mutant background.

The following fly strains were also used: elav-mCD8-GFP [48], FRT2A, UAS-mCD8-GFP, dpp^blink^-GAL4 [78], Sqh::EYFP-Golgi [43], UAS-nβgal, hs-Flp, P{GawB}ey^OK107^, PBac{GAL4D, EYFP}GlcAT-P^PL00294^ (used at GlcAT-P-GAL4), PBac{PB}CG14142^c00688^, PBac{PB}^c01618^, *807-GAL4* (A Brand, unpublished) and Tb^1^. Fly stocks without reference are described in flybase. Unless otherwise specified, all the mutant analysis was done in brv^1^ mutant allele. GlcAT-P cDNA was amplified from DGRC plasmid LD40245 by PCR using the oligos RP1 (AATTGCGGCCGCTTGGTTCGTCACAATTTTTTATA) and RP2 (AATTGGTACCTTTTTTTTTTTTTTTTTTGGAAAATG). Catalytically dead GlcAT-P was generated by mutating Asp333 to Ala by PCR. For making respective UAS-GlcAT-P lines, the PCR products were cloned into the NotI-KpnI cleaved transformation vector pUAST-attB.

### Mapping the EMS lesions

RNA was extracted from homozygous *brv* mutant larvae using the RNAeasy kit (Qiagen). cDNAs derived from the 10 genes in the mapped region were synthesized using standard protocols (Invitrogen) and sequenced for all the three alleles.

### Rescue constructs and EGFP tagging by recombineering

The following construct from P[acman] resource (see http://flypush.imgen.bcm.tmc.edu/pacmanfly) was used for rescue experiments: *CH322-82E19* (contains *GlcAT-P* without the first two exons). N or C terminus of GlcAT-P was tagged to EGFP in *CH322-82E19* and *CH321-38B20* (contains entire *GlcAT-P* and some other genes) BACs from P[acman] resource. We used Addgene plasmids 19173 and 19178 for EGFP-tagging by recombineering [45]. All germ line transformants were generated by Bestgene Inc, CA.

### Antibody generation

Polyclonal antibodies were generated against peptides C-RTRYKNTNLEHIDRLLVRP and C-EGRNALISKNGRENPHSK for GlcAT-P and GlcAT-S respectively (Genzym Antibodies).

### Immunoblotting

Third instar larvae or their brains were used for extract preparation. Protein extracts were resolved by SDS-PAGE. Immunoblots were probed with anti-HNK-1 (1∶1000), rabbit anti-GlcAT-P (1∶1000), rabbit anti-GlcAT-S (1∶1000) and mouse anti-α-tubulin (1∶10000, Sigma) using PVDF membranes (Perkin Elmer) and Supersignal-detection kit (Thermo Scientific).

### Immunohistochemistry

The following primary antibodies were used: mouse anti-elav (1∶20, Developmental Studies Hybridoma Bank (DSHB)), mouse anti-repo (1∶5, DSHB), mouse anti-antp (1∶20, DSHB), mouse anti-AbdB (1∶10, DSHB), mouse anti-Ubx (1∶20, kindly provided by R White), rat anti-AbdA (1∶200, kindly provided by J Casanova), mouse anti-HNK-1 (1∶1000, kindly provided by S Oka), rabbit anti-Mira (1∶5000, kindly provided by F Matsuzaki). In addition, secondary goat antibodies conjugated to Alexa488 (Molecular Probes), Cy3 or Cy5 (Jackson Immuno Research Laboratories) were applied. Images were acquired with an Upright Olympus FV-1000 Confocal Imaging system using FV10-ASW software.

For releasing the connections of the posterior peripheral nerves with the larval muscles, the appropriately staged wild-type and *brv* mutant larvae carrying *elav-mCD-GFP* were first cold-immobilized. An image was captured using Leica MZ16F stereomicroscope before and after the posterior-most nerve connections were separated by making a minute cut in the posterior part of the larvae.

## Supporting Information

Figure S1 Molecular characterization of *brv^1^*, *brv^2^* and *brv^3^* alleles. Partial alignments of the cDNA (A, C, D) and genomic DNA (B) sequences from *brv^+^*, *brv^1^*, *brv^2^,* and *brv^3^* alleles are presented. The 14 bp long deletion in the *brv^1^* cDNA (boxed) is a consequence of a G to A mutation in the splice acceptor (black box) and the use of a cryptic alternative splice acceptor (red box). A G to A mutation transforms a Trp codon (TGG) to a Stop codon (TAG) (C, boxed) in *brv^2^*. A T to A transversion changes the conserved, non-polar, Leu residue (CTG) into the polar Gln amino acid (CAG) (D, boxed) in *brv^3^*. (E) Partial alignment of several GlcAT-P orthologs shows that the *brv^3^* mutation affects an evolutionarily conserved hydrophobic residue (boxed).
(TIF)

Figure S2 (A) Protein preparations from *brv^+^* and *Df(3L)GlcAT-P* brains were blotted with anti-HNK-1 (HNK-1) and anti-GlcAT-S (GlcAT-S) antibodies. (B–C) Misexpression of *UAS-GlcAT-P* in L3 larva wing imaginal discs with *Dpp^blink^-GAL4.* (B) Ectopic expression of *GlcAT-P* is detected by α-GlcAT-P immunohistochemistry in the typical pattern of *dpp* expression domain. The white box indicates the region shown in (C). (C) Co-labeling (arrows) of α-GlcAT-P *(*red) *with Sqh::EYFP-Golgi* (green) reveals that GlcAT-P protein is localized to the Golgi apparatus. Bars in B and C are 100 µm and 20 µm respectively.
(TIF)

Figure S3 *GlcAT-P-GAL4* driven expression of a reporter gene (nuclear β-Gal) recapitulates aspects of endogenous *GlcAT-P* expression during embryogenesis. (A, B) Note that reporter gene expression mimics the described *GlcAT-P* in situ hybridization expression pattern (see BDGP;http://www.fruitfly.org/cgi-bin/ex/bquery.pl?qtype=report&find=CG6207&searchfield=CG). LacZ expression was detected in the amnioserosa (A; arrows) of stage 14 embryos, and in salivary glands (B; arrowheads) and the gut (arrows) at stage 17. Bar: 100 µm.
(TIF)

Figure S4 The general structure the VNC is unaffected in *brv* mutant larvae. Labeling of L3 larva brains from wild-type (*brv^+^*) and *brv^1^* with anti-22C10 (A, B), anti-BP102 (C, D), anti-Fas2 (E, F), anti-Elav (G, H) and anti-Repo (I, J) shows apparently normal VNC structure in *brv* mutants. Bar: 50 µm.
(TIF)

Figure S5 Mushroom body neurons and VUM motoneurons are apparently normal in *brv* mutants. (A, B) *OK107-GAL4>UAS-mCD8-GFP* did not display any apparent defects in mushroom bodies in L3 brains. Arrowheads point to the MB neurons. (C, D) *807-GAL4*>*UAS-mCD8-GFP* showed that VUM neurons were present in *brv^1^* mutants (arrowheads). Two consecutive abdominal neuromeres (dashed lines) are shown. A–D represent maximum projections of Z-stacks. Bar: 20 µm.
(TIF)

Figure S6 Peripheral glia spacing and distribution is not affected in *brv* mutant larvae. (A) Labeling of L3 larva brains from *brv^+^* and *brv^1^* mutants with anti-Repo antibody shows a similar spacing between glia in the peripheral nerves. (B) *Nrv2-GAL4>GFP* expression in L3 larva brains from wild-type (*brv^+^*) and *brv^1^* mutants. (C) Merged frames of anti-Repo (red) and *Nrv2-GAL4>GFP* (green). (D) Histograms depicting the average number of glial cells in comparable stretches of the most posterior peripheral nerves of *brv^+^* and *brv^1^* (n = 30 for each) larvae. Bar: 50 µm.
(TIF)

Figure S7 Rescue of *brv* mutants by expression of *GlcAT-P* using UAS/GAL4 system. Quantification of the length of the L3 VNCs (A) and peripheral nerves (B) in different rescue experiment settings is provided (*: p<0.0001).
(TIF)
