# Supplementary figures and images for: The Glucuronyltransferase GlcAT-P Is Required for Stretch Growth of Peripheral Nerves in Drosophila
Source: PLoS One. 2011 Nov 23;6(11):e28106. doi: 10.1371/journal.pone.0028106 (PMC3223219; doi:10.1371/journal.pone.0028106)

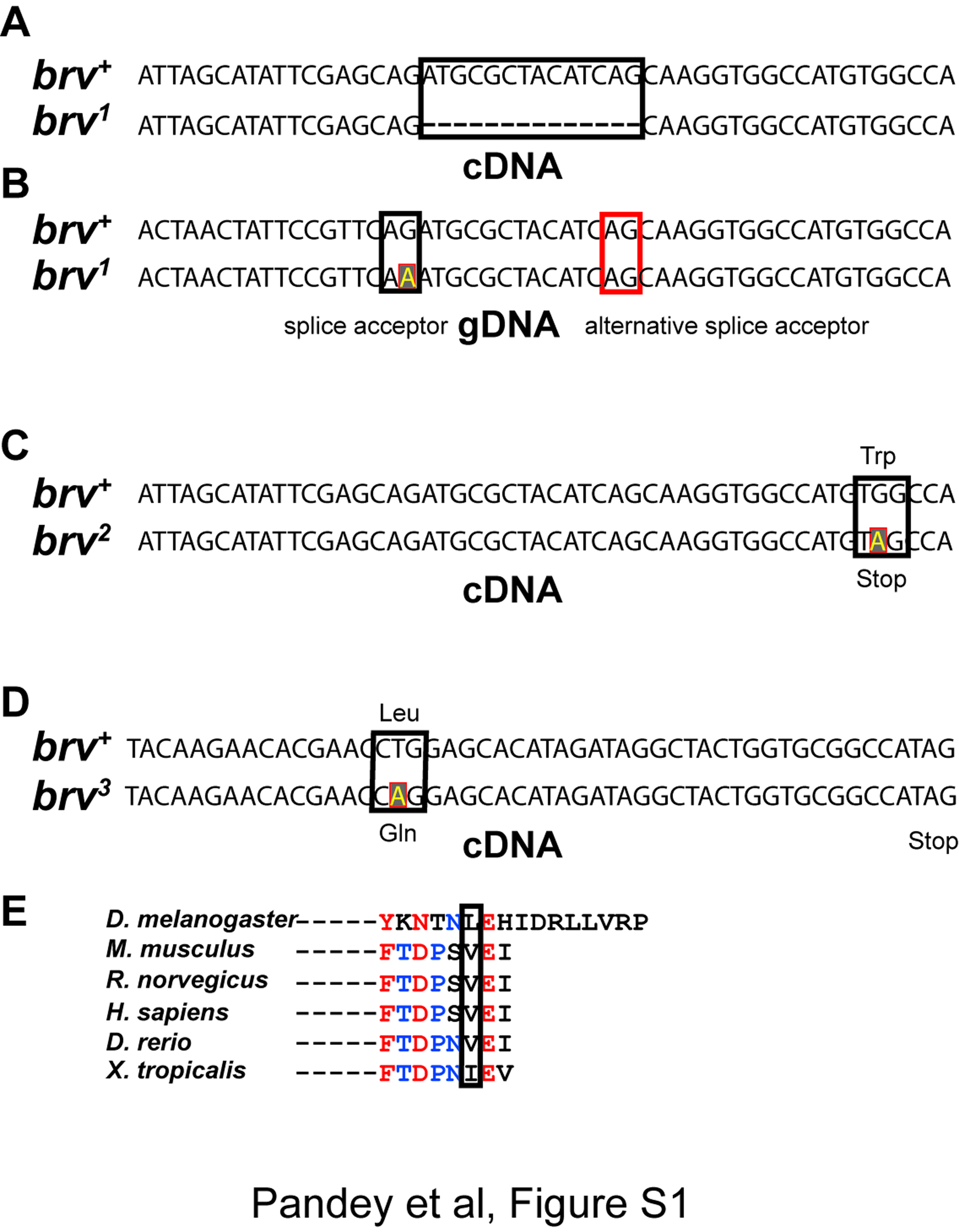

Supplement: Figure S1 — Molecular characterization of brv1, brv2 and brv3 alleles. Partial alignments of the cDNA (A, C, D) and genomic DNA (B) sequences from brv+, brv1, brv2, and brv3 alleles are presented. The 14 bp long deletion in the brv1 cDNA (boxed) is a consequence of a G to A mutation in the splice acceptor (black box) and the use of a cryptic alternative splice acceptor (red box). A G to A mutation transforms a Trp codon (TGG) to a Stop codon (TAG) (C, boxed) in brv2. A T to A transversion changes the conserved, non-polar, Leu residue (CTG) into the polar Gln amino acid (CAG) (D, boxed) in brv3. (E) Partial alignment of several GlcAT-P orthologs shows that the brv3 mutation affects an evolutionarily conserved hydrophobic residue (boxed). (TIF) [file pone.0028106.s001.tif]

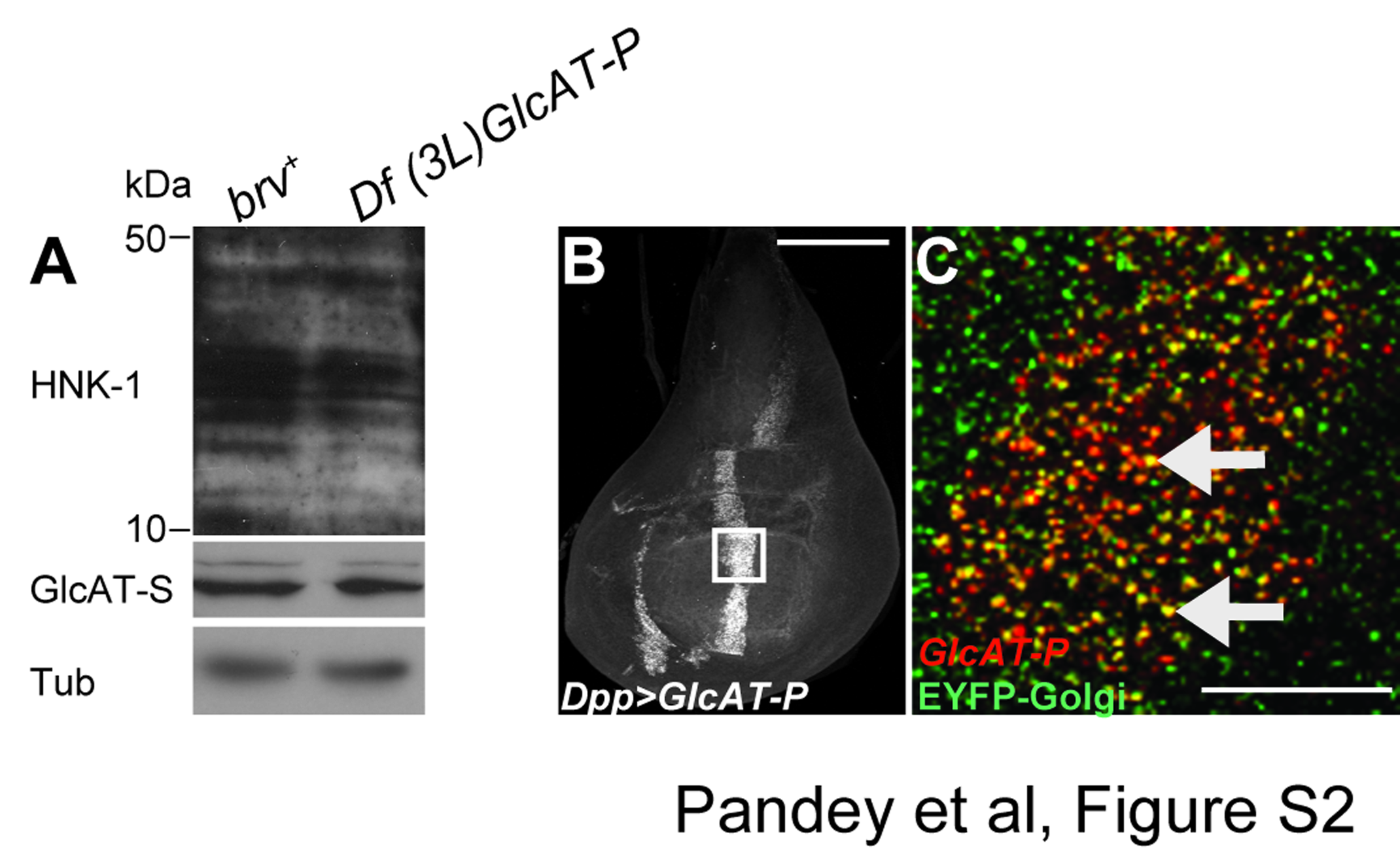

Supplement: Figure S2 — (A) Protein preparations from brv+ and Df(3L)GlcAT-P brains were blotted with anti-HNK-1 (HNK-1) and anti-GlcAT-S (GlcAT-S) antibodies. (B–C) Misexpression of UAS-GlcAT-P in L3 larva wing imaginal discs with Dppblink-GAL4. (B) Ectopic expression of GlcAT-P is detected by α-GlcAT-P immunohistochemistry in the typical pattern of dpp expression domain. The white box indicates the region shown in (C). (C) Co-labeling (arrows) of α-GlcAT-P (red) with Sqh::EYFP-Golgi (green) reveals that GlcAT-P protein is localized to the Golgi apparatus. Bars in B and C are 100 µm and 20 µm respectively. (TIF) [file pone.0028106.s002.tif]

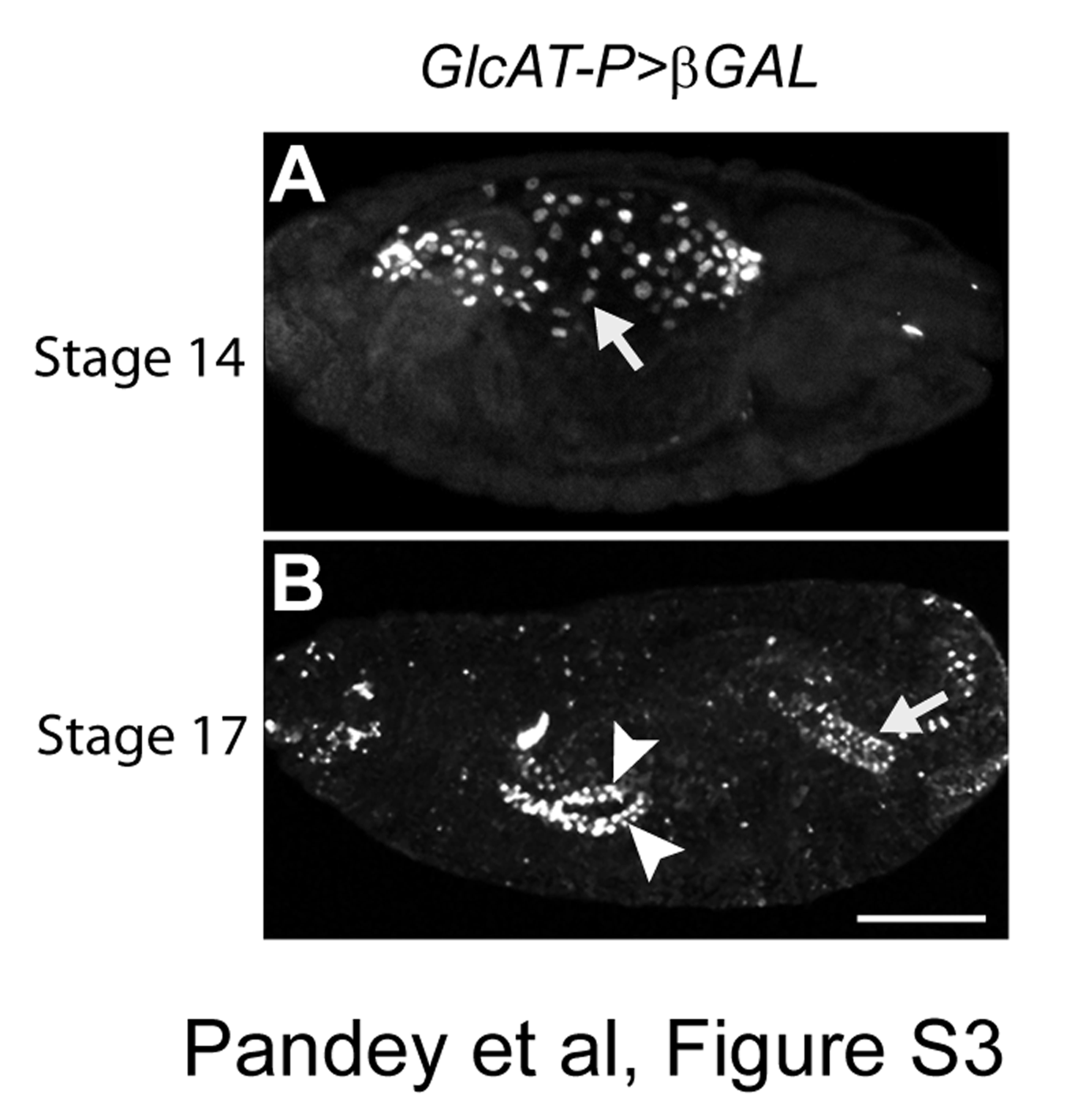

Supplement: Figure S3 — GlcAT-P-GAL4 driven expression of a reporter gene (nuclear β-Gal) recapitulates aspects of endogenous GlcAT-P expression during embryogenesis. (A, B) Note that reporter gene expression mimics the described GlcAT-P in situ hybridization expression pattern (see BDGP;http://www.fruitfly.org/cgi-bin/ex/bquery.pl?qtype=report&find=CG6207&searchfield=CG). LacZ expression was detected in the amnioserosa (A; arrows) of stage 14 embryos, and in salivary glands (B; arrowheads) and the gut (arrows) at stage 17. Bar: 100 µm. (TIF) [file pone.0028106.s003.tif]

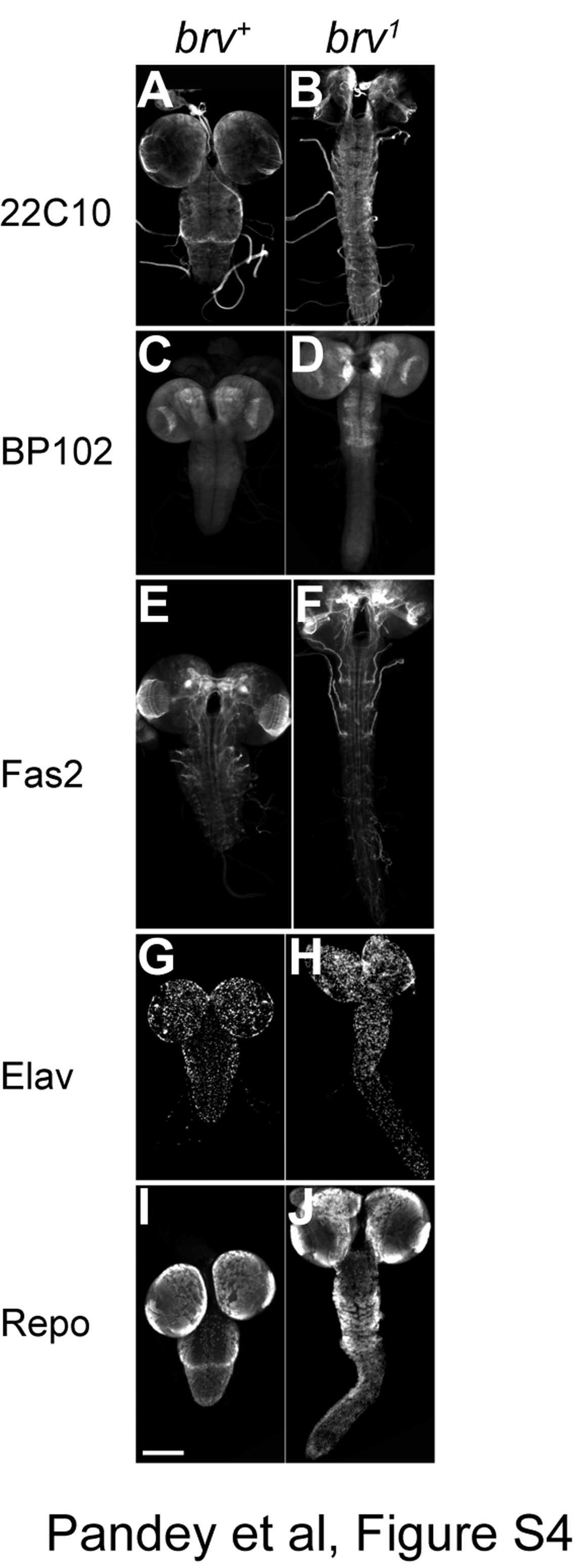

Supplement: Figure S4 — The general structure the VNC is unaffected in brv mutant larvae. Labeling of L3 larva brains from wild-type (brv+) and brv1 with anti-22C10 (A, B), anti-BP102 (C, D), anti-Fas2 (E, F), anti-Elav (G, H) and anti-Repo (I, J) shows apparently normal VNC structure in brv mutants. Bar: 50 µm. (TIF) [file pone.0028106.s004.tif]

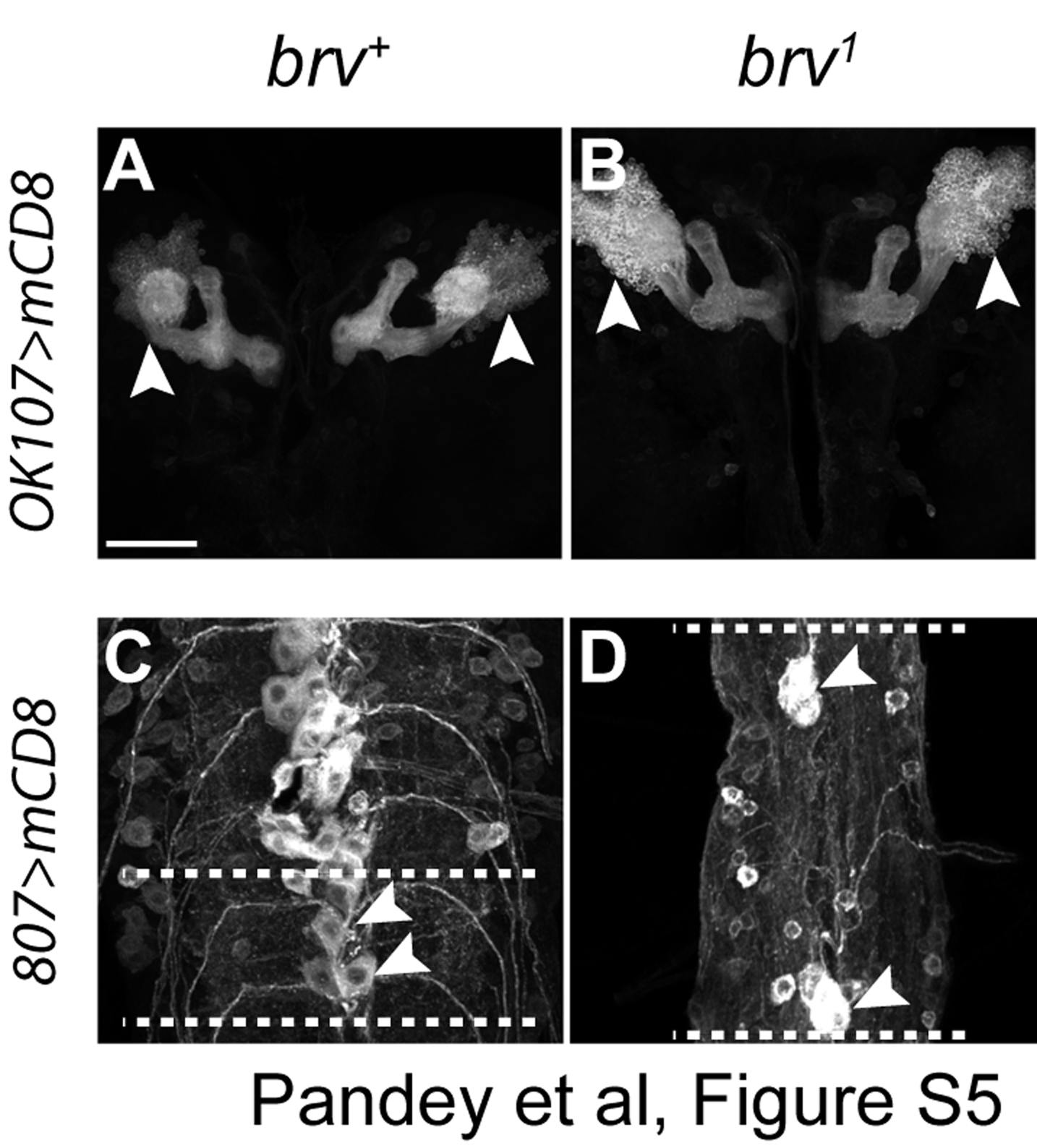

Supplement: Figure S5 — Mushroom body neurons and VUM motoneurons are apparently normal in brv mutants. (A, B) OK107-GAL4>UAS-mCD8-GFP did not display any apparent defects in mushroom bodies in L3 brains. Arrowheads point to the MB neurons. (C, D) 807-GAL4>UAS-mCD8-GFP showed that VUM neurons were present in brv1 mutants (arrowheads). Two consecutive abdominal neuromeres (dashed lines) are shown. A–D represent maximum projections of Z-stacks. Bar: 20 µm. (TIF) [file pone.0028106.s005.tif]

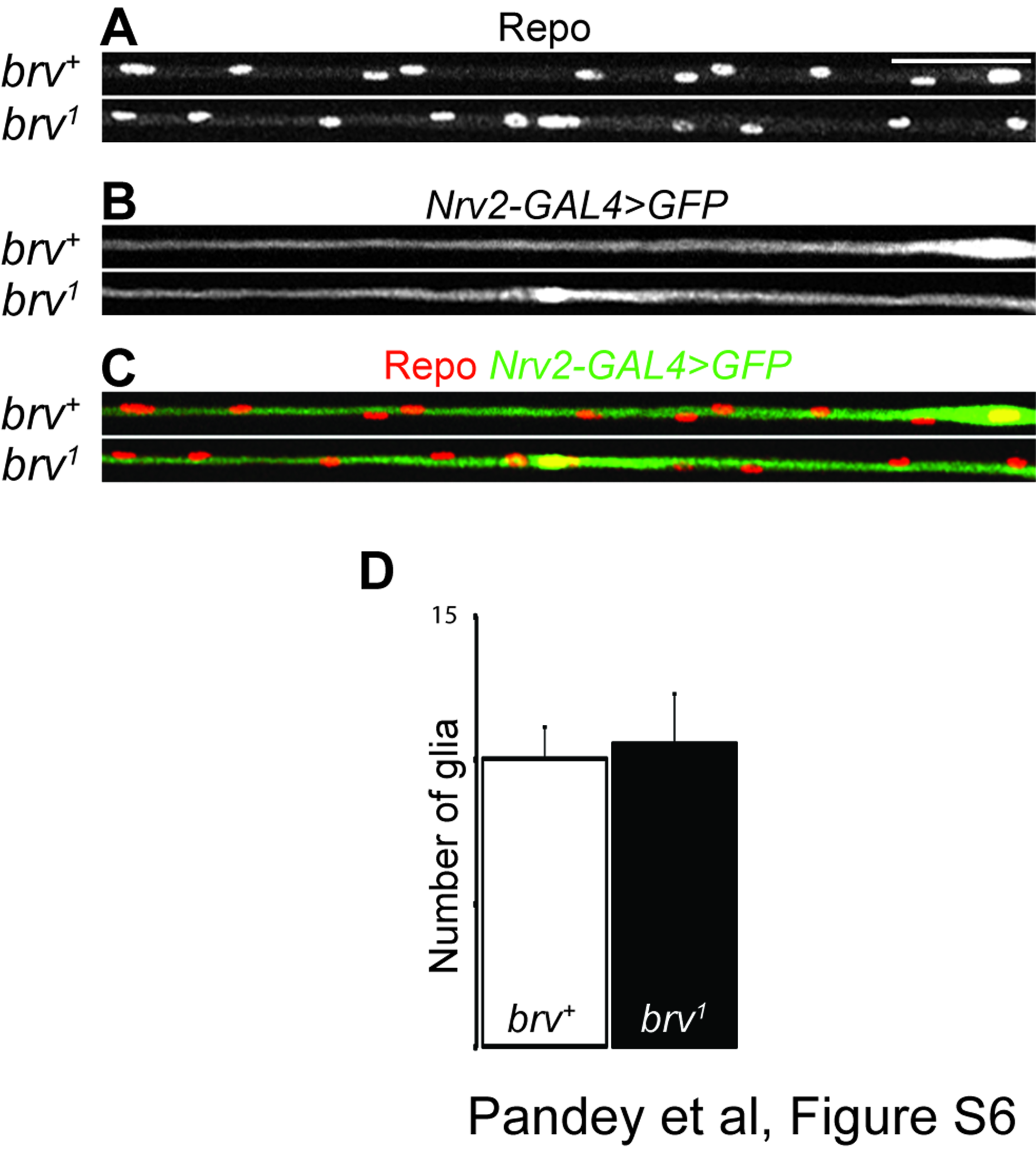

Supplement: Figure S6 — Peripheral glia spacing and distribution is not affected in brv mutant larvae. (A) Labeling of L3 larva brains from brv+ and brv1 mutants with anti-Repo antibody shows a similar spacing between glia in the peripheral nerves. (B) Nrv2-GAL4>GFP expression in L3 larva brains from wild-type (brv+) and brv1 mutants. (C) Merged frames of anti-Repo (red) and Nrv2-GAL4>GFP (green). (D) Histograms depicting the average number of glial cells in comparable stretches of the most posterior peripheral nerves of brv+ and brv1 (n = 30 for each) larvae. Bar: 50 µm. (TIF) [file pone.0028106.s006.tif]

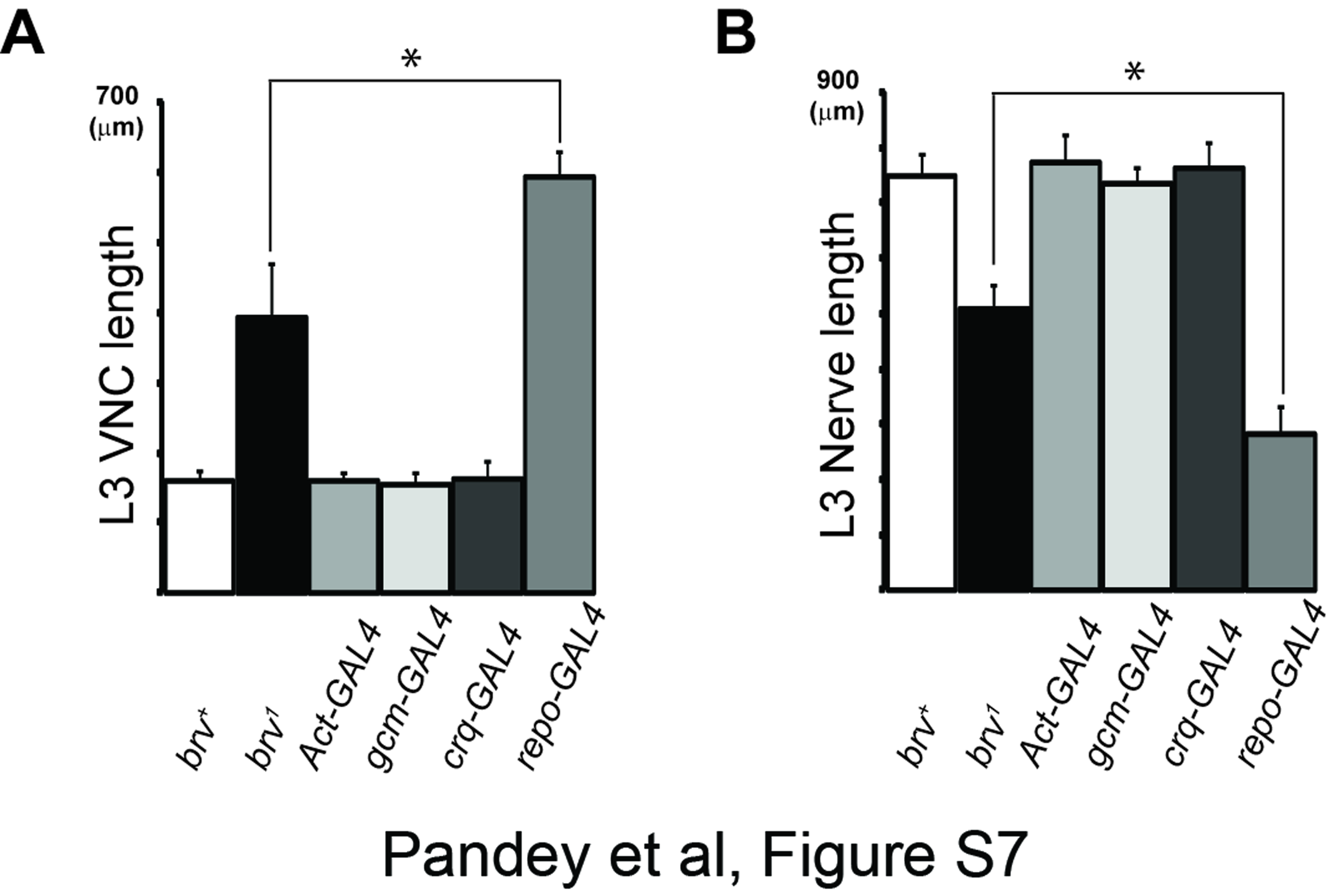

Supplement: Figure S7 — Rescue of brv mutants by expression of GlcAT-P using UAS/GAL4 system. Quantification of the length of the L3 VNCs (A) and peripheral nerves (B) in different rescue experiment settings is provided (*: p<0.0001). (TIF) [file pone.0028106.s007.tif]
